# Are Covert Saccade Functionally Relevant in Vestibular Hypofunction?

**DOI:** 10.1007/s12311-017-0907-0

**Published:** 2017-12-16

**Authors:** R. Hermann, D. Pelisson, O Dumas, Ch Urquizar, E. Truy, C. Tilikete

**Affiliations:** 1 Hospices Civils de Lyon, ENT, Cervico-Facial Surgery and Audiophonology, Hôpital Edouard Herriot, Lyon, France; 2 INSERM, U1028, CNRS, UMR5292, Lyon Neuroscience Research Center, IMPACT Team, Lyon, France; 3 Lyon I University, Lyon, France; 4 French Vestibular Rehabilitation Society, Lyon, France; 5 Hospices Civils de Lyon, Neuro-Ophthalmology Unit, Hopital Neurologique et Neurochirurgical P Wertheimer, Lyon, France

**Keywords:** Vestibular areflexia, Bilateral vestibulopathy, Dynamic visual acuity, Head impulse test, Oscillopsia, Eye movement record

## Abstract

The vestibulo-ocular reflex maintains gaze stabilization during angular or linear head accelerations, allowing adequate dynamic visual acuity. In case of bilateral vestibular hypofunction, patients use saccades to compensate for the reduced vestibulo-ocular reflex function, with covert saccades occurring even during the head displacement. In this study, we questioned whether covert saccades help maintain dynamic visual acuity, and evaluated which characteristic of these saccades are the most relevant to improve visual function. We prospectively included 18 patients with chronic bilateral vestibular hypofunction. Subjects underwent evaluation of dynamic visual acuity in the horizontal plane as well as video recording of their head and eye positions during horizontal head impulse tests in both directions (36 ears tested). Frequency, latency, consistency of covert saccade initiation, and gain of covert saccades as well as residual vestibulo-ocular reflex gain were calculated. We found no correlation between residual vestibulo-ocular reflex gain and dynamic visual acuity. Dynamic visual acuity performance was however positively correlated with the frequency and gain of covert saccades and negatively correlated with covert saccade latency. There was no correlation between consistency of covert saccade initiation and dynamic visual acuity. Even though gaze stabilization in space during covert saccades might be of very short duration, these refixation saccades seem to improve vision in patients with bilateral vestibular hypofunction during angular head impulses. These findings emphasize the need for specific rehabilitation technics that favor the triggering of covert saccades. The physiological origin of covert saccades is discussed.

## Introduction

In humans, different precise and well-coordinated eye movements facilitate a clear and stable vision of the environment by directing the fovea toward an object of interest, then stabilizing the image of this target on the fovea (foveation). When exploring a visual scene, the image of an object of interest has to be brought within 0.5° from the fovea to ensure optimal visual acuity [1]. This is accomplished by fast and precise eye movements called saccades. The fixation system then comes into play to stabilize the eyes relative to a stable object. In case of moving object or moving subject relative to the environment, stabilizing eye movements such as smooth pursuit, optokinetic reflex, and vestibulo-ocular reflex are required. The goal of these eye fixation and stabilization responses is to limit retinal slip or movement of images on the retina, as visual acuity steeply decreases when retinal slip exceeds 4°/s [2]. Foveation is therefore maintained while the head is motionless as well as during rotational or translational head movements despite the latter reaching speeds of up to 550°/s during daily life activities [3]. However, visually-guided stabilizing eye movements, such as smooth pursuit or optokinetic reflex, cannot keep up in case of such high-velocity head rotations. Instead, the vestibulo-ocular reflex (VOR), which responds to changes in head acceleration with a latency of 5–7 ms [4, 5], is the only efficient eye movement for stabilizing gaze in this condition. In case of vestibular deficit, besides postural deficits, patients may complain of decreased visual acuity during head motion and of oscillopsia—an illusion of an unstable visual world—during head movements. These symptoms can have a severe impact on the quality of life of patients with bilateral vestibular hypofunction (BVH) [6]. To compensate for their deficient VOR, patients with BVH use two different types of saccades to redirect their fovea toward the object of interest when they move their head. The first type, called overt saccades (OS) [7], occurs after the head movements and are thus likely elicited by visual information about the target of interest. The second type, called covert saccades (CS) [8], occurs during the on-going head movement. Whereas OS can be clinically identified with the head impulse test (HIT) [7], the demonstration of CS requires recording of eye movements during the HIT. The latency of CS can be as short as 70 ms [9] which is shorter than the 100-ms latency of express saccades [10]. Some sparse case reports suggest that CS are associated with a better visual function and quality of life in patients with BVH [11, 12]. The few controlled studies questioning the impact of CS suggest that vestibular disability and handicap, tested with Dizziness Handicap Inventory, is lower when CS show small variability in latency [13, 14]. One study has highlighted the importance of covert saccade frequency to account for better dynamic visual acuity (DVA) performance, but the patients only had a unilateral vestibular hypofunction and gain, and latency of CS were not analyzed [15]. The aim of our study was to evaluate in patients with BVH the functional visual impact of CS tested by DVA. This study further questioned which characteristic of these saccades—frequency, latency, consistency of latency or gain—could be the most relevant to improve visual function.

## Methods

### Study Population

This prospective study was held in the neuro-ophthalmology unit in University Hospital in Lyon between April 2016 and January 2017. All patients underwent neurological and vestibular assessment. The inclusion criteria were (1) age between 18 and 90; (2) bilateral vestibular hypofunction shown with caloric water irrigation (bilateral and bithermal [30/44 °C], median peak slow phase velocity of 5°/s or less), rotatory chair test (burst and step in the horizontal plane, VOR gain lower than 0.2), and HIT (VOR gain lower than 0.6); and (3) stability of BVH over at least 6 months. Patients with a corrected visual acuity lower than 0.3 LogMAR, other lesions leading to ataxia and/or oscillopsia, oculomotor palsy or ocular instability in primary gaze position, and patients with instability of the cervical spine were not included.

Eighteen patients, 9 men and 9 women, were included in our study. The median age was 64 (range 22–80) with no difference between men and women (respectively 60 years [SD 15] and 59.6 years [SD 19.2]).

### Ethical Issue

All patients were informed about the design and purpose of the study, and all gave their informed, written consent to the protocol. Approval was received from the National French ethical committee on human experimentation (n°160165B-32), in agreement with French law (March 4, 2002) and the Declaration of Helsinki. The study was registered in a public trials registry (ClinicalTrials.gov Identifier NCT02753179).

### Functional Visual Evaluation

Standard visual acuity was first evaluated with best eye correction using a Monoyer chart. Static and dynamic visual acuities were then evaluated using a commercialized device (Framiral, Grasse, France), as described in the following.

#### Static Visual Acuity

Visual acuity was first measured statically using Sloan letter optotypes flashed on a computer screen. The subject sat at a distance of 80 cm in front of the screen and had to read a letter that was flashed three times for 50 ms with a 1-s interval between each flash. The size of the first letter corresponded to a visual acuity of 0.3 logMAR. The subject gave his answer after having read the same flashed letter three times. The examiner reported the success/failure of the answer by clicking respectively on the left or right pad of the computer mouse. The Quest algorithm was used to determine the change in size of the next optotype. The starting function was $\frac{1}{\sigma \sqrt{2\pi }}{e}^{\frac{1}{\sigma \sqrt{2\pi }}}$ (with *σ* (standard deviation) = 0.2 and *μ* (location parameter) = 0.05) and the function for success or failure was the Weibull function ${W}_{T(x)}=1-{e}^{\left[-\left(\frac{x+\varepsilon }{\alpha}\right)\beta \right]}$ (with *α* (scale parameter) = 0.5, *β* (shape parameter) = 5, and *ε* (location parameter) = 0.53). The test kept going until the standard deviation of the starting function was inferior to 0.05.

#### Dynamic Visual Acuity

**Fig. 1:**
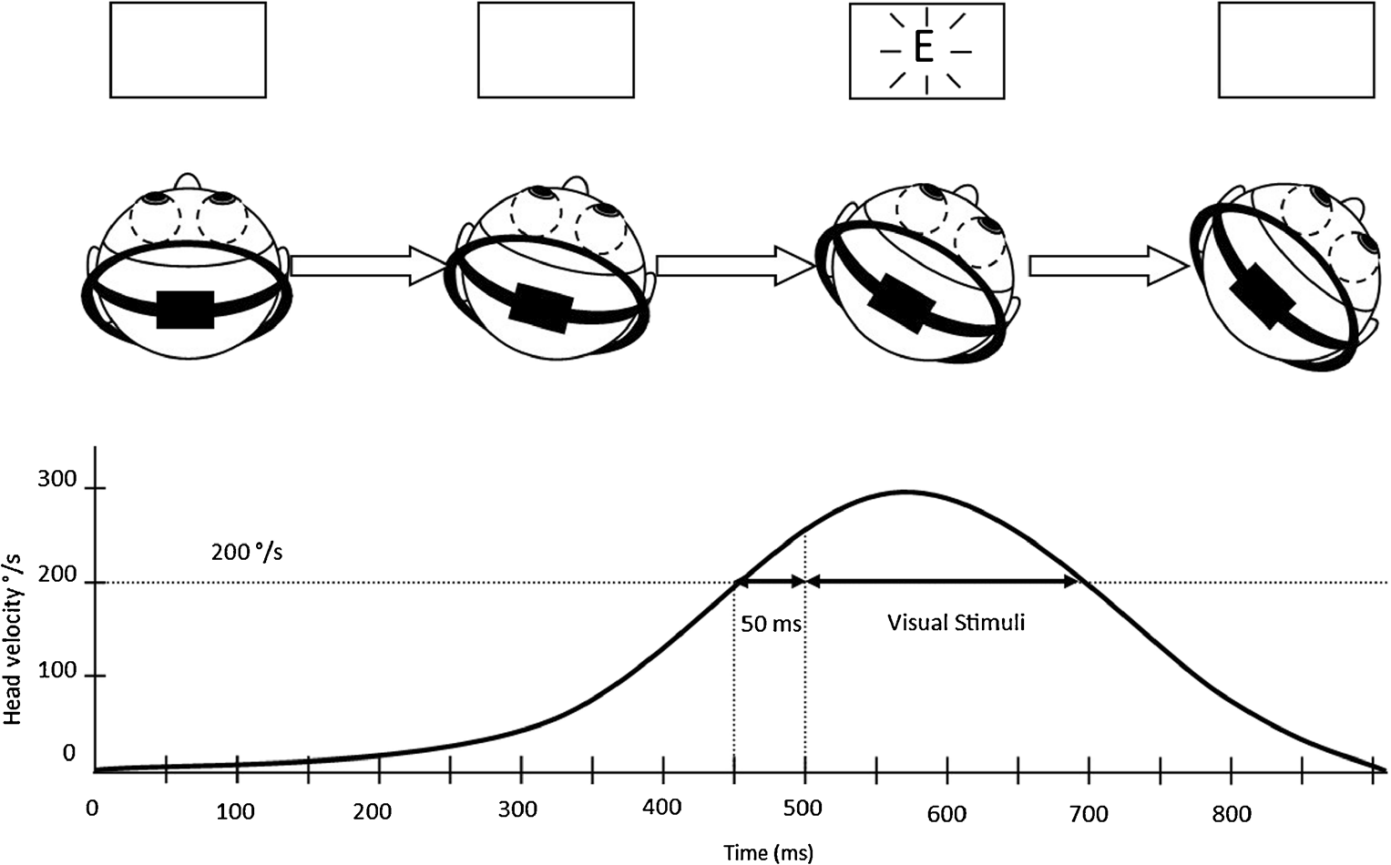
Schematic representation of change in head position and relative head velocity with time during measurement of the dynamic visual acuity. The optotype (letter “E” in this example) appears on the screen only when head rotation velocity is within a range of 200 to 300°/s for more than 50 ms (“Visual Stimuli”)

For measures of dynamic visual acuity, the subjects wore a lightweight helmet equipped with a nine-axis motion tracking device recording head velocity at a 100-Hz frequency. Subjects were asked to look at the center of the screen prior to the onset of the optotype. The visual stimuli were enslaved to head rotations and only appeared if the velocity of the head rotation was within a 200 to 300°/s range for more than 50 ms (Fig. 1). An audible tone was played when the head rotation velocity was within the expected range for more than 50 ms. Subjects could train beforehand to initiate the correct head movements. Subjects were then asked to perform three active head impulses for a given stimulus before answering. DVA was measured in lateral directions of head rotations with the same method used for static visual acuity. Left and right DVA were tested separately. This test was done with eyeglasses if needed.

Static and dynamic visual acuity was given in LogMAR. Since visual performance is inversely proportional to LogMAR measures, we will refer to performance for the correlation analyses.

### Eye and Head Movements Recording During Head Impulse Tests

vHIT was performed 15 min after DVA testing. The patients were seated at a distance of 1.5 m from a visual fixation target placed at eye level. They wore a lightweight portable vHIT device (Hardware—ICS Impulse, GN Otometrics, Taastrup, Denmark, Software—Otosuite® Vestibular software) to record their eye and head movements during head impulse tests. Head movements were recorded with a nine-axis motion sensor, and movements of the right eye were monitored with a high-velocity infrared camera, both the sensor and camera being mounted on a lightweight eye frame. The sampling rate was 250 Hz for both eye and head movements. The eye movement calibration was performed by having the subject gaze toward two built-in lasers projecting at eye level on the wall with the head still. Calibrated eye position data was then derived by the abovementioned software to obtain eye velocity data.

#### Head Impulse Test

Centrifugal horizontal head impulses were performed by the examiner standing behind the patient. The head was tilted forward to align the plane of the horizontal canals with the horizontal plane. Horizontal head impulses were performed in each direction. Valid head impulses had to be above 200°/s and free from major artifacts. A minimum of 10 and a maximum of 20 valid head impulses were recorded by the Otosuite program. Head and eye velocity data were then exported in Cascading Style Sheets (CSS) format for off-line analysis.

#### Analysis of Head and Eye Movements

Eye and head positions were calculated by integrating velocity signals over time with the following formula:

$$ {\displaystyle \begin{array}{l}\mathrm{Eye}\kern0.5em \mathrm{Position}\kern0.5em \left(P\mathrm{t}\right)=0.004\kern0.5em \times \kern0.5em \frac{\mathrm{Eye}\kern0.5em \mathrm{speed}\kern0.5em \left( Pt-1\right)+\kern0.5em \mathrm{Eye}\kern0.5em \mathrm{speed}(Pt)}{2}+\mathrm{Eye}\kern0.5em \mathrm{position}\left({P}_{t-1}\right)\\ {}\left(\mathrm{considering}\kern0.5em \mathrm{that}\kern0.5em {P}_0=0\right)\kern0.5em \left( Pt\kern0.5em =\mathrm{actual}\kern0.5em \mathrm{eye}\ \mathrm{position}, Pt-1=\kern0.5em \mathrm{previous}\ \mathrm{eye}\ \mathrm{position},0.004\kern0.5em =\kern0.5em \mathrm{time}\  \mathrm{between}\ \mathrm{two}\ \mathrm{consecutive}\ \mathrm{eye}\ \mathrm{position}\  \mathrm{samples}\right)\end{array}} $$

**Fig. 2:**
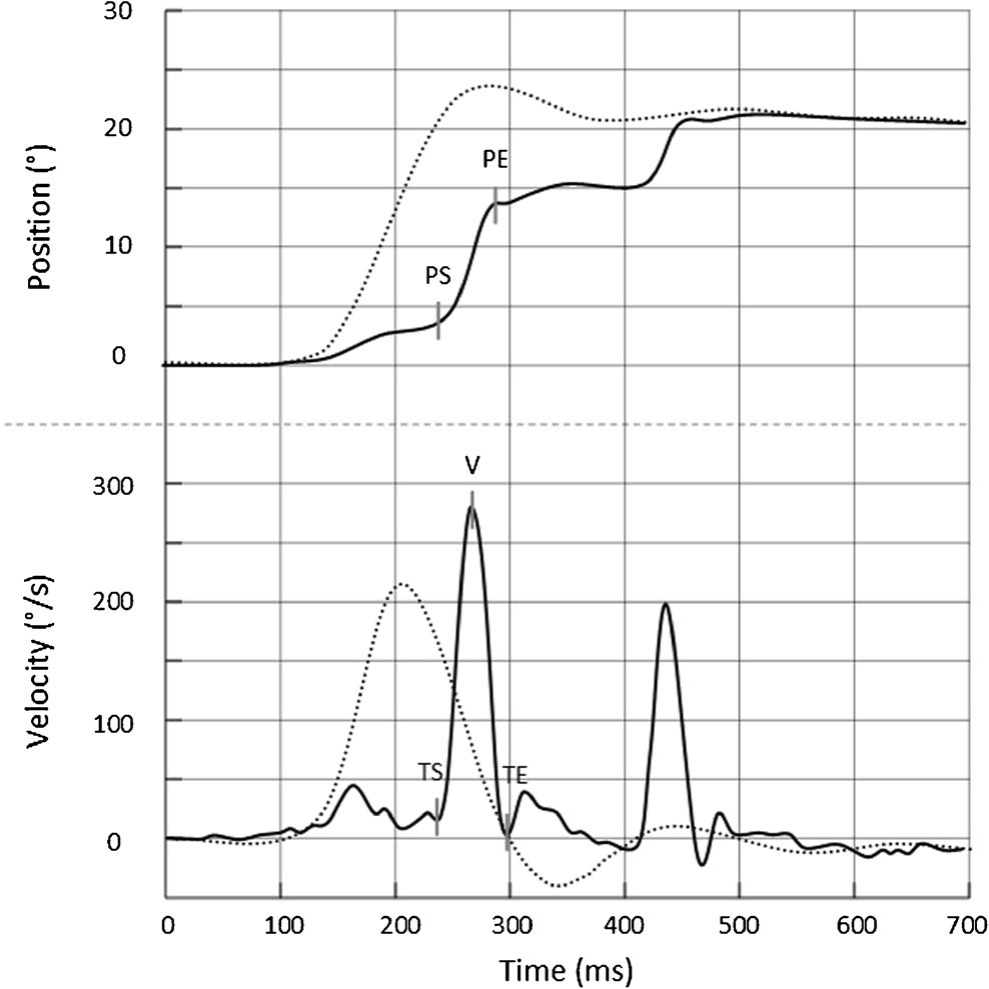
Example of cursor placements on a covert saccade. Eye (^_____^) and head (_·········_) position and velocity traces for a head impulse test in a patient. The head position and velocity waveforms are inverted relative to eye position and velocity waveforms. The vertical cursors represent the starting position (PS) and ending positions (PE), the time of starting position (TS) and the time of ending position (TE) of the movement, and the maximum velocity of the movement (V)

Head and eye position and velocity were then analyzed in a program developed in our lab running on MATLAB v.8.1 (MathWorks, MA, USA). The graphical interface of this software shows eye and head position and velocity trajectories (Fig. 2). For each eye movement and for the head movement, five different cursors are automatically placed at the starting and ending time, the starting and ending position and the maximum velocity. All cursors were checked and manually adjusted if necessary. Slow eye movements occurring between the beginning and the end of the head movement (in one direction) or between the beginning of the head movement and the occurrence of the first saccade were considered as a VOR sequence. The end of the VOR sequence was hence defined as either the start of the covert saccade (by visually identifying the break point of the eye velocity curve [see Fig. 2 cursor TS]) or the maximum point of VOR amplitude in case of absence of covert saccades. Saccades were identified by the abrupt change in eye velocity and eye position profiles, better seen by overlaying velocity and position data. A saccade beginning before the end of the head movement was considered as a covert saccade while a saccade beginning after head movement termination was considered as an overt saccade. Amplitudes for covert and overt saccades were defined as maximum amplitude of theses saccades.

Two measures of head movement onset were carried out: after the beginning of head movement when head velocity reached 5°/s or after the beginning of significant retinal slip when head velocity reached 30°/sec.

Different parameters were calculated from these data:

- For each head impulse
  - VOR gain was calculated as (amplitude of eye movement during VOR sequence)/(amplitude of head movement at the end of VOR sequence).
  - Saccadic gain was calculated as (amplitude of eye movement during CS or OS)/(amplitude of total head movement).
  - Latencies were calculated as the onset time of each eye movement (VOR, CS, OS) relative to the beginning of head movement (hm_latency) or to the beginning of significant retinal slip (rs_latency).
- For each subject
  - Occurrence of CS was determined in percent as the total number of CS relative to the total number of head impulses.
  - Consistency of CS initiation was determined by the standard deviation of individual (for each ear) standard deviation of CS rs_latency.

### Statistical Analysis

All data were stored and analyzed using Statistica 10 (Softonic®, Barcelona, Spain). We analyzed the correlations between the different parameters of CS (frequency, gain, latency, and variability of latency) and DVA measures. Statistical analysis was done using Spearman’s rank order correlation as well as linear regression and Mann-Whitney’s *U* test. *p* values < 0.05 were considered significant.

## Results

### Overall Results

The main features of the 18 subjects are shown in Table 1.

**Table 1:** General data of all patients

*N°*	Gender	Age	Duration (years)	Etiology	VOR	SVA (cor)
1	M	77	1	Iatrogenic—gentamycin	0.13	0
2	M	65	7	Idiopathic	0.18	0
3	F	63	32	Iatrogenic—gentamycin	0.18	0
4	F	69	2	Bilateral vestibular neuritis	0.17	0
5	M	58	2	Iatrogenic—gentamycin	0.04	0
6	M	68	10	Idiopathic	0.19	0
7	M	51	4	Idiopathic	0.09	0
8	M	74	10	Iatrogenic—gentamycin	0.01	0.1
9	F	41	31	Cochleo-vestibular hypofunction	0.06	0
10	F	79	5	Type II neurofibromatosis	0.03	0.1
11	F	80	9	Idiopathic	0.03	0
12	M	47	11	Post infectious	0.01	0
13	F	52	2	Idiopathic	0.04	0
14	F	62	16	Meniere’s disease	0.06	0.05
15	M	31	4	Idiopathic	0.2	0
16	M	72	12	Idiopathic	0.02	0.1
17	F	72	15	Idiopathic	0	0.1
18	F	22	3	Type II neurofibromatosis	0.04	0

Gender: *F*, female; *M*, male. *VOR*, vestibulo-ocular reflex gain during rotatory chair testing; *SVA (cor)*, corrected static visual acuity with Monoyer test in LogMAR; *Cochleo-vestibular hypofunction*, progressive BVH associated with progressive profound bilateral hearing loss

### Visual Acuity

Median standard best corrected visual acuity measure with Monoyer test was 0.0 LogMAR (interquartile range (IR) 0.03). Four subjects used progressive spectacles during the tests. Median flashed static visual acuity measure was 0.18 LogMAR (IR 0.16). The median dynamic visual acuity measure was 0.49 LogMAR (IR 0.25). Median dynamic visual acuity performance was statistically lower than median static visual acuity performance (*p* < 0.001).

### Eye and Head Movements During Head Impulse Tests

vHIT was performed in all patients. The left and right sides were analyzed separately for a total of 36 tested inner ears. The median amplitude of head movements during vHIT tests was 21.16° (IR 2.53°), and their median duration was 150.18 ms (IR 14 ms).

CS were present for all but one tested inner ear and occurred in 76% of head impulses.

**Fig. 3:**
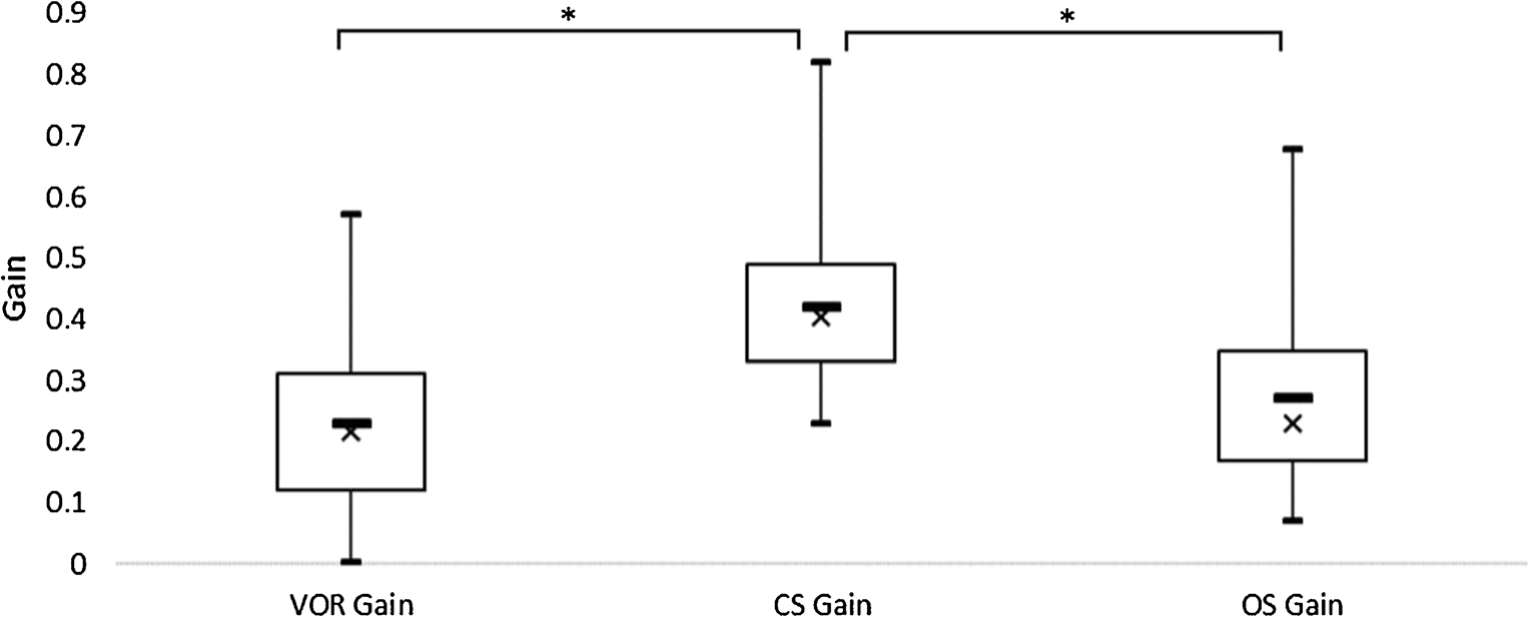
Boxplots of the gain of the vestibulo-ocular reflex (VOR), of covert saccades (CS), and of overt saccades (OS) during head impulse testing showing lower extreme, 1st quartile, median (cross), median (larger horizontal line), 3rd quartile, and upper extreme. Statistical differences (*) are considered significant with a *p* < 0.05

The median residual VOR gain during vHIT was 0.22 (IR 0.19). The median gain of CS and OS was respectively 0.40 (IR 0.16) and 0.23 (IR 0.18). Median CS gain was higher than median residual VOR gain (*p* < 0.001) and median OS gain (*p* < 0.001) (Fig. 3).

The median CS hm_latency and rs_latency were respectively 145 ms (IR 36.38) and 120 ms (SD 30.91). The shortest CS rs_latency was 69 ms and hm_latency 91 ms (individual value). The median consistency of CS rs_latencies was 13.06 ms (IR 5.95). The median OS rs_latency was 259 ms (IR 62).

### Statistical Correlation Between DVA, Eye Movements, and Age

Since visual performance is inversely proportional to LogMAR measures, we will refer to performance for the correlation analyses.

DVA performance was significantly and positively linked with frequency of occurrence of CS (*R* ^2^ = 0.25; *p* < 0.005).

DVA performance was also positively correlated with CS gain (*R* ^2^ = 0.42; *p* < 0.001) but not with VOR gain (*p* = 0.39).

**Fig. 4:**
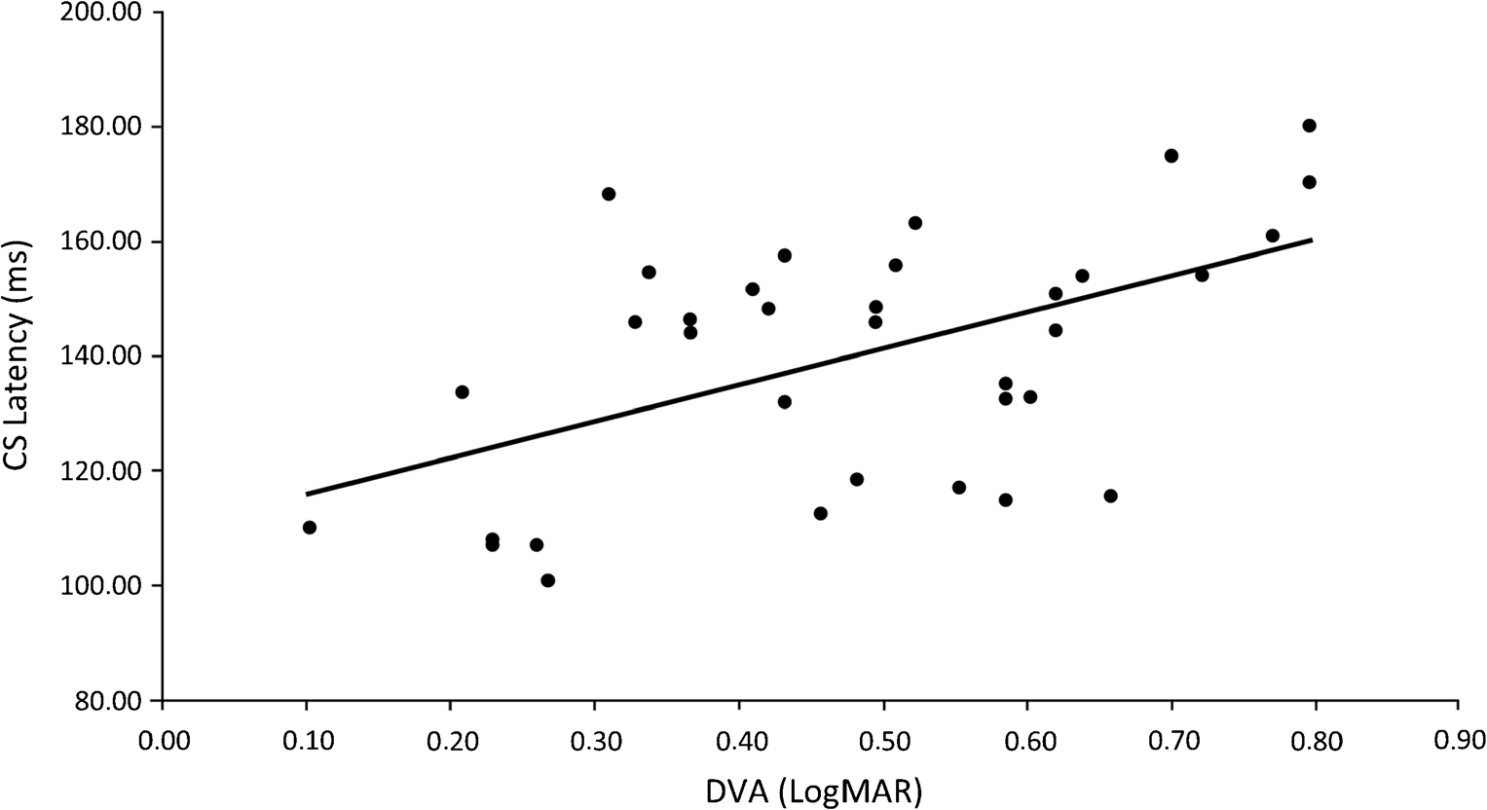
Linear regression of CS rs_latency and DVA measures

DVA performance was significantly inversely correlated with CS rs_latency (*R* ^2^ = 0.23; *p* < 0.005) (i.e., better visual performance associated with shorter latencies, Fig. 4) and correlated with OS rs_latency (*R* ^2^ = 0.14; *p* < 0.05).

There was no significant correlation between CS rs_latency and OS rs_latency (*p* = 0.93).

We found no correlation between the consistency of CS initiation and DVA (*p* = 0.85), even in a selected population of ears (*n* = 29) with high CS occurrence (> 50%) (*p* = 0.89).

There was no statistical link between residual VOR gain and CS frequency (*p* = 0.52), CS latency (*p* = 0.91), and CS gain (*p* = 0.62).

Age was significantly inversely correlated to DVA performance (*R* ^2^ = 0.59; *p* < 0.001) (worse visual performance with increasing age), CS frequency (*R* ^2^ = 045; *p* < 0.001), CS gain (*R* ^2^ = 0.27; *p* = 0.001), and positively linked to CS rs_latency (*R* ^2^ = 0.2; *p* < 0.01) and OS rs_latency (*R* ^2^ = 0.14; *p* < 0.05) (i.e., DVA performance was worse, CS gain and frequency were lower and CS rs_latency longer in older patients) but not to residual VOR gain (*p* = 0.38)). We found no significant correlation between duration of symptoms and VOR or CS variables.

## Discussion

The goal of this study was to evaluate in patients with bilateral vestibular hypofunction (BVH) the potential functional impact of covert saccades (CS) on visual performance. To our knowledge, this is the first study evaluating dynamic visual acuity (DVA) and vHIT data in subjects with bilateral vestibular hypofunction. We analyzed compensatory eye movements during head impulses and found a significant link between DVA and CS parameters (frequency of occurrence, gain, and latency) which suggests that CS help to improve vision during head movement.

When comparing to other studies [16, 17], our population was representative of patients suffering from BVH considering etiologies as well as age and sex ratio. In our population of BVH, CS occurred in 76% of head impulses. The eye movements performed to stabilize the gaze on the target during and after the head impulse test were an association of residual VOR, covert saccade, and overt saccade which where respectively responsible on average for 23, 42, and 27% of the total eye movement. CS represented thus the largest eye displacement, accounting for nearly half of the total eye displacement compensating head movement. These results are close to previous data suggesting that CS reduced gaze error by an average of 37% [9]. The median CS hm_latency was 140 ms, and the single shortest CS was 91 ms in our study, being around 20 ms longer than the shortest CS latencies found in the literature [8, 9]. The median latency for the first catch-up saccade (overt and covert) was 152 versus 135 ms in the literature [8]. This could be due to the difference in population (uni- versus bilateral vestibular deficit), choice of head velocity threshold, the timing for the onset of the CS (change in velocity and position versus peak acceleration), and the method of eye and head movement recording (scleral coil versus video recording and nine-axis motion sensor). These data, emphasizing the amount and amplitude of CS in BVH, further justify to examine the functional role of CS for vision during head movements.

In cases of bilateral VOR impairment, gaze stabilization during high-velocity head motion is insufficient to allow a clear and stable vision of the environment. This can be evaluated using visual acuity measure during active head impulse. In our population, median DVA measure was 0.49 LogMAR. DVA performance has already been shown to be impaired in vestibular areflexia, although the exact values show a great variability amongst studies (0.83 to 0.31) due to differences in method and populations [15, 18]. In our study, the amount of residual VOR gain, ranging from 0 to 0.57 at high frequencies, is not linked with DVA. While DVA appears to be a good indicator of visual consequences of vestibular impairment, it does not seem to be an accurate indicator of the amount of VOR impairment.

On the other hand, our study suggests that covert saccades may compensate the impaired vestibular eye movement to improve vision during head thrusts. Indeed, our results indicate that DVA performance improves significantly with frequency of occurrence and gain of CS. Furthermore, the shorter the CS latency the better the DVA performance. A previous study in unilateral vestibular areflexia has already highlighted the critical contribution of covert saccades to DVA, as CS frequency was linked to DVA performance, but gain and latency of CS were not analyzed [15]. Earlier studies have also highlighted the functional impact of consistency of CS initiation on vestibular instability and handicap (reflected by the DHI scale) [13, 14]. However, in our study, we did not find any correlation between the consistency of CS initiation and DVA. We finally suggest that covert saccades could help stabilize gaze during a foveation period sufficiently well to allow flashed optotype reading during high-velocity head movements. This foveation period might be obtained if saccade velocity can negate head velocity for a sufficient period.

As already shown in healthy subjects [19], we found a significant influence of age in decreased DVA performance. In healthy subjects, DVA performance that declines with aging can be attributed both to physiological vestibular and visual system aging [19]. We also found a decreased CS occurrence and gain as well as an increased CS latency with age, which could be attributed to physiological aging of saccadic eye movements [20] and/or decreased neural plasticity needed for the development of compensatory eye movements in case of vestibular areflexia. Our study does not allow us to tease apart the relative contribution to DVA performance of aging of the visual system versus age-related decrease of CS efficiency; a multivariate analysis in a larger cohort of patients would be necessary to address this issue.

In our study, we evaluated functional visual processing using DVA. However, one of the main complaints of patients suffering from BVH is oscillopsia, the illusion of an unstable visual world due to a lack of gaze stabilization. Although loss of DVA performance and oscillopsia often occur together as they are both a consequence of an impaired VOR, no study has tried to bring to light a direct correlation between decreased DVA performance and oscillopsia in patients with already impaired VOR. Oscillopsia is a subjective consequence of retinal slippage, and it has been shown that a higher tolerance to retinal slippage was correlated with lower oscillopsia handicap score [20]. Moreover, people with BVH have been shown to have diminished visual motion processing at high head velocities [21], suggesting that they learn to tolerate retinal slip more than normal subjects. One could also suggest that CS may participate in lowering oscillopsia in BVH since during saccades, perception of retinal slip is suppressed [22]. A study evaluating oscillopsia score and CS could test these hypotheses.

Our study confirms that in BVH, saccadic eye movements compensate deficient slow vestibular ones. Saccades can also compensate for other deficient slow eye movements such as catch-up saccades observed in deficient smooth pursuit. While during deficient smooth pursuit, catch-up saccades are triggered by the moving visual target, the trigger of covert saccades in BVH remains unanswered. Previous data showed that the amplitude of compensatory saccades in patients with BVH was related to the amount of head displacement, suggesting that amplitude of saccades is based on calculation of a gaze position error signal [9]. Gaze position error based on residual vestibular signals has been suggested [9], but our data show no correlation between CS efficiency (occurrence, gain, and latency) and residual VOR gain. Visually calculated gaze position error, based on retinal slip at the beginning of head movement, is another possibility. Since short latencies of about 70 ms have been already observed in the ocular following response [23], retinal drift could also trigger covert saccades through this optokinetic pathway. However, the observation of CS in darkness [9] would argue against retinal slip as the sole trigger of saccades. Cervically (neck proprioception), calculation of gaze position error and triggering of CS could be another possibility. Indeed, cervically triggered compensatory saccades have been observed in one patient with BVH [24]. However, the latency of such saccades is not known. Internally triggered saccades, calculated in anticipation of head movement of different amplitudes thanks to some period of learning, is a last possibility. This initiation mode could explain why CS are better triggered during active versus passive head movements [25]. A study evaluating development of CS following acute loss of vestibular function could be helpful to differentiate between these different hypotheses.

In our study, we deliberately decided to investigate active head movements for the DVA and passive movements for the vHIT. vHIT were performed passively in order to comply with the technique described by MacDougall et al. and to be able to compare our results with those of the literature. Furthermore, we wanted all head impulses to have similar characteristics (direction, amplitude, speed) which were not possible with active head impulses. On the other hand, we wanted DVA evaluation to be a functional measure, tested in the most physiological context, a reason why we chose active head impulses. Since it seems that active rotation increases the occurrence of covert saccades [11, 25], using different head movement conditions for DVA and vHIT could have led to underestimate covert saccade occurrence during vHIT.

## Conclusion

Our study highlights the role of covert saccades to improving dynamic visual acuity in bilateral vestibular hypofunction. CS parameters playing a significant role in DVA performance improvement include a higher occurrence rate, a shorter latency, and a higher gain. These findings emphasize the need for specific rehabilitation technics that focus on triggering covert saccades.
